# Surgical treatment of slipped capital femoral epiphysis (SCFE) by Dunn procedure modified by Ganz: a systematic review

**DOI:** 10.1186/s12891-022-05071-9

**Published:** 2022-02-07

**Authors:** Giulio Gorgolini, Alessandro Caterini, Kristian Efremov, Lidio Petrungaro, Fernando De Maio, Ernesto Ippolito, Pasquale Farsetti

**Affiliations:** Department of Clinical Sciences and Translational Medicine. Division of Orthopaedic Surgery, University of Rome “Tor Vergata”, Viale Oxford 81, 00133 Rome, Italy

**Keywords:** Slipped capital femoral epiphysis, SCFE, Dunn osteotomy, Ganz surgical approach, Surgical hip dislocation, Flip trochanter osteotomy

## Abstract

**Background:**

Treatment of SCFE is still controversial, especially in moderate and severe forms. Dunn osteotomy performed with the Ganz approach became very popular in the last decade, although it is a complicated and challenging surgical procedure with a risk of AVN. The aim of our study was to analyze the current literature verifying the effectiveness of this surgical procedure, with specific attention to the incidence of AVN and other complications.

**Main body:**

A systematic review on the subject was performed according to the PRISMA guidelines. A literature search was performed by searching all published articles about the topic in the databases. The articles were screened for the presence of the following inclusion criteria: patients affected by slipped capital femoral epiphysis (SCFE) surgically treated by Dunn osteotomy using the Ganz surgical approach. All the patients affected by pathologies other than SCFE, treated without surgery or with procedures not including a surgical hip dislocation were excluded.

Based on inclusion and exclusion criteria, 23 studies were included in our systematic review. Selected articles were published from 2009 to 2021 and they included 636 overall hips. According to the selected articles, Dunn osteotomy modified by Ganz, performed by an experienced surgeon, allows for anatomical reduction of moderate or severe SCFE with a low incidence of AVN.

**Conclusions:**

The few papers with long term follow-up, reported no progression of hip osteoarthritis, however, since the patients are adolescent at surgery, longer follow-up studies are needed to validate this statement. It is still debated if better results are obtained in stable or unstable SCFE. The indication of this procedure in mild SCFE remains controversial.

**Level of evidence:**

3

## Background

Slipped capital femoral epiphysis (SCFE) occurs with an overall incidence of 10.8 cases/100.000 children (1:7500 in males and 1:12.500 in females), between 9 and 16 years of age, with an average age of 13 years in males and 11.8, in females; it may be bilateral in 18 to 50% of the cases [1, 2]. Its etiology remains uncertain, but histological, histochemical and ultrastructural changes have been reported in the pathogenesis of this disease [3, 4].

SCFE is commonly classified depending on the duration of the clinical symptoms in acute (up to 3 weeks), chronic (more than 3 weeks) and acute on chronic, and in stable and unstable based on the walking capacity of the affected patient [5, 6]. In stable hips, the patient is able to bear weight with or without crutches, while in unstable hips, weight bearing is not possible even with crutches [6]. It is also classified from a radiographic point of view in three types, according to the severity of the posterior displacement of the capital epiphysis, which is measured using the Southwick angle; SCFE is defined mild when the Southwick angle measures < 30°, moderate when the angle measures between 30° to 60° and severe when it is > 60° [7].

The classic treatment for acute or acute on chronic SCFE, generally unstable, was represented by an attempt of gentle closed reduction followed by a fixation in situ, while for the chronic forms, usually stable, the most common surgical treatment is represented by in situ pinning or by triplane trochanteric osteotomy, such as the Imhauser or Southwick osteotomy [7, 8]. Both these techniques, especially in moderate or severe forms, did not restore the anatomy of the femoral head and often femoro-acetabular impingement (FAI) occurs with premature hip osteoarthritis.

Dunn in 1964 first described a surgical technique for SCFE with the primary goal to “replace the femoral head on the end of the neck without stretching the retinacular vessels” [9]. In the original paper, he reported 19 good results out of 23 SCFE and four complications, two avascular necrosis (AVN) and two condrolysis. Several years later, Dunn and Angel better described the surgical technique that they performed in a cohort of 73 SCFE (25 acute on chronic and 48 severe chronic) [10]. They used a postero-lateral approach, performing the capsule incision along the axis of the femoral neck and extended it round the anterior and posterior edge of the acetabulum. The authors never dislocated the femoral head but with extreme care detached it from the femoral neck which was shortened; the head was anatomically reposition on the shortened neck and stabilized with three pins. The authors observed a clinical-radiological fair or poor result in 8–25% of the chronic slip and in 30–36% of acute on chronic slips and concluded that open reduction is an excellent treatment for severe chronic slipping in patients with open physis and that the main complications are observed in acute on chronic forms; they also speculated that the damage of the blood supply of the femoral head in these cases occurred at the time of the acute slip, before surgical reduction [10].

In 2001 Ganz et al. [11] first described a modification of the Dunn technique “with full access to the femoral head and acetabulum without risk of AVN”. The operation consisted of an anterior dislocation of the hip based on detailed studies published by the same authors a year earlier on the vascular anatomy of the hip [12]. The authors, through a posterior/postero-lateral hip approach, performed a “trochanteric flip” osteotomy and a z-shaped capsulotomy anterior to the lesser trochanter, to preserve the deep branch of the medial femoral artery. They reported 213 surgical dislocations of the femoral head with various indications in patients with a mean age of 33.5 years, without a single case of AVN. The technique of surgical dislocation presented in their study allows a visualization of the entire femoral head and a complete access to the acetabulum, therefore in the subsequent years it was adopted by many orthopedic surgeons for approaching the hip joint in skeletally immature patients affected by SCFE.

The present study is a systematic review in which we analyzed all the available literature published after the description of the “surgical hip dislocation technique”, reporting the clinical and radiological results with the incidence of AVN of the femoral head, in patients affected by SCFE surgically treated by Dunn osteotomy using the Ganz surgical approach. The aim of this study was to verify the effectiveness of this surgical procedure for the different types of SCFE, with specific attention to the incidence of AVN that in adolescent patients represents a dramatic complication only resolved through the application of a total hip prosthesis at a young age.

## Main text

We performed a systematic review on the subject according to the PRISMA guidelines [13, 14]. Inclusion and exclusion criteria were formulated according to the PICO method [15] and they were summarized in Table 1.

**Table 1:** Inclusion and exclusion criteria (PICO)

	Inclusion criteria	Exclusion criteria
**Population**	- Patients affected by slipped capital femoral epiphysis (SCFE)	- Patients who did not underwent surgery
**Intervention**	- Dunn osteotomy modified by Ganz with surgical hip dislocation approach	- Surgical techniques without hip dislocation - Non-surgical treatment
**Comparison group**	- Studies reporting patients affected by SCFE treated by Dunn procedure modified by Ganz, including comparative studies with in situ pinning or Imhauser osteotomy	- Not applicable
**Outcome**	- Studies reporting clinical and radiographic scores	- Not applicable
**Time**	- Studies published from 2001 to 2021	- Studies published prior to 2001
**Study type**	- Clinical Trials - Cohort Studies - Observational Studies - Randomized Control Trials	- Letters - Case reports - Case series < 10 hips
**Language**	- English	- Other languages

Search strategy and sources of information:

Authors of this review (GG, AC, KE, FDM, PF) performed a literature search about the topic by querying Medline database. Studies were located by searching the databases. The search strategy covers PICO and was performed independently by each author in February 2021. Keywords and MeSH Terms were identified by a preliminary search and selected by discussion. The search was conducted using the following keywords and their synonyms, assembled in various combination to obtain the most pertinent articles: Slipped capital femoral epiphysis, SCFE, Dunn osteotomy, Ganz surgical approach, surgical hip dislocation.

The following search query were used: (((“slipped capital femoral epiphyses”[MeSH Terms] OR (“slipped”[All Fields] AND “capital”[All Fields] AND “femoral”[All Fields] AND “epiphyses”[All Fields]) OR “slipped capital femoral epiphyses”[All Fields] OR (“slipped”[All Fields] AND “capital”[All Fields] AND “femoral”[All Fields] AND “epiphysis”[All Fields]) OR “slipped capital femoral epiphysis”[All Fields]) OR (“Slipped Capital Femoral”[Title/Abstract]) OR (“Slipped Femoral”[Title/Abstract]) OR (“SCFE”[Title/Abstract])) AND ((“GANZ”[Title/Abstract]) OR (“Dunn”[Title/Abstract]) OR (“Southwick”[Title/Abstract]))) OR ((“southwick”[All Fields] OR “southwick s”[All Fields]) AND (“osteotomie”[All Fields] OR “osteotomied”[All Fields] OR “osteotomy”[MeSH Terms] OR “osteotomy”[All Fields] OR “osteotomies”[All Fields])) OR ((“ganz”[All Fields] AND (“osteotomie”[All Fields] OR “osteotomied”[All Fields] OR “osteotomy”[MeSH Terms] OR “osteotomy”[All Fields] OR “osteotomies”[All Fields]) AND ((“slipped capital femoral epiphyses”[MeSH Terms] OR (“slipped”[All Fields] AND “capital”[All Fields] AND “femoral”[All Fields] AND “epiphyses”[All Fields]) OR “slipped capital femoral epiphyses”[All Fields] OR (“slipped”[All Fields] AND “capital”[All Fields] AND “femoral”[All Fields] AND “epiphysis”[All Fields]) OR “slipped capital femoral epiphysis”[All Fields]) OR (“Slipped Capital Femoral”[Title/Abstract]) OR (“Slipped Femoral”[Title/Abstract]) OR (“SCFE”[Title/Abstract])))) OR (“dunn”[All Fields] AND (“osteotomie”[All Fields] OR “osteotomied”[All Fields] OR “osteotomy”[MeSH Terms] OR “osteotomy”[All Fields] OR “osteotomies”[All Fields])) OR (“dunn procedure”[All Fields]) OR (“modified dunn osteotomy”[All Fields]).

Publication date filter was applied to select only articles from 2001 since it was the year in which Ganz et al. described for the first time their surgical approach for Dunn osteotomy. Language restriction were applied to identify only English articles.

The reviewers (GG, AC, KE, FDM, PF) retrieved the data and independently analyzed each selected study; instances of disagreement were resolved by the senior investigator (PF).

The articles were screened for the presence of the following inclusion criteria:

1. Patients affected by slipped capital femoral epiphysis (reporting at least 10 cases with a minimum follow-up of 1 year)
2. Patients surgically treated by Dunn osteotomy modified by Ganz with surgical hip dislocation technique
3. Studies with different level of evidence, including retrospective studies.
4. Availability of full text
5. Studies published from 2001 to 2021

The articles were excluded if any of the following exclusion criteria were identified:

1. Diagnostic or prognostic studies
2. Non-surgical treatment of patients
3. Other surgical approaches (in situ pinning, peritrochanteric osteotomy, Dunn osteotomy without hip dislocation)
4. Studies non pertinent with SCFE
5. Full text in a different language than English
6. Studies published prior to 2001

**Fig. 1:**
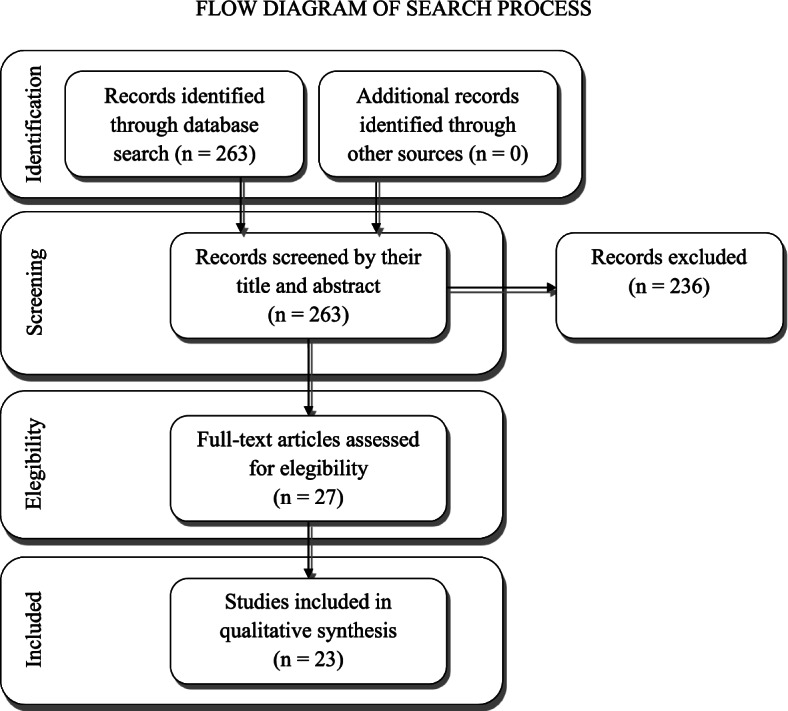
Flow diagram, describing the number of studies identified, included and excluded with relative reasons

Figure 1 shows the flowchart for study selection.

The initial search produced 257 studies from Medline database, 18 studies from Scopus and 7 from WoS, for a total of 282 studies. Of the 25 studies found on Scopus and WoS, 19 were duplicates, while 6 were new unique entries. All records were screened by their title and abstract and 236 studies were excluded since they did not meet our inclusion criteria. They were excluded for the following reasons: 109 reported a not pertinent topic, 109 reporting a pertinent topic but did not meet all the inclusion criteria of the study design, 18 articles were published in a language different than English.

After detailed evaluation of the full text of the remaining 27 studies, based on inclusion and exclusion criteria, 23 papers were selected to be include in the present review [16–38]; 4 articles fulfilled the inclusion criteria but reported fewer than 10 cases.

Selected articles were published from 2009 to 2021 and they included 636 hips overall, with a follow up that ranged between 1.2 and 12 years. Of these, 399 were stable, while the remaining 237 unstable. Based on the onset, 174 hips were considered acute, 221 chronic, and 164 acute-on-chronic; 77 were not classified. Based on the slip angle, 29 hips were classified as mild (< 30°), 97 as moderate (30°-60°) and 339 as severe (60°-90°); one paper with 21 hips reported the mean slip angle of 59.1°, but not how many were in each category; this information was not available for the remaining 150 hips. The incidence of AVN was variable, with some studies reporting no cases of AVN, to some with a high incidence of up to 29.4% of AVN. A total of 69 cases of AVN were reported, with a mean incidence of 10.8%. Other complications such as implant failure, heterotopic ossifications and limb length inequality were reported with various frequencies.

Of the 23 studies included in our review, most of them reported satisfactory results. Results were summarized and classified with validated scales and scores in 18 studies. One study reported results without a validated scale or score, while 4 studies did not report classified outcome values.

The most commonly used validated scales and scores reported to describe outcomes were Harris Hip Score (HHS) and The Merle d’Aubigné Hip Score (MdA), but in some papers results were reported using UCLA activity score, Hip Disability and Osteoarthritis Outcome Score (HOOS), Western Ontario and McMaster University score (WOMAC), Nottingham Health Profile score (NHP), Visual Analog Scale (VAS), and Heyman and Herdon score.

Fourteen studies reported an HHS with a mean value of 91,95 (range 76.3–99.6), 7 studies reported an MdA score with a mean value of 16.98, 6 studies reported a Womac score with a mean value of 96,2. Three studies reported Heyman and Herdon excellent or good scores in 60, 67% and 97,3% of cases respectively. Two studies reported a NAHS of 85.4 and 91.3. Finally, 1 last study reported satisfactory results in 94% of cases. Four studies did not report a classification of outcomes with outcome values.

Table 2 presents the list of reference of the studies, type of study, number of cases, average age at surgery, classifications, length of follow-up, results, incidence of AVN and other complications and conclusions.

**Table 2:** Characteristics, data, results and conclusions of the included studies

Paper	Number of Hips	Age at surgery (average)	Classification:stable/ unstable	Classification: acute/chronic/acute-on-chronic	Classification: mild/moderate/ severe	Lenght of Follow-Up (average)	Results	Incidence of AVN	Other complications	Conclusions
Agashe et al., Indian J Orthop, 2021 [38] (Retrospective)	30	13.0 y	19/11	6/6/18	0/20/10	2.1 y	Average HHS: 81.8	6.6%	Hip subluxation: 3.3%	Procedure safe, reliable and reproducible; first choice for treatment of moderate and severe SCFE.
Passaplan et al., Bone Joint Open, 2020 [37] (Retrospective)	18	12.9 y	14/4	1/3/14	8/8/2	9.4 y	Average HHS: 88.7; HOOS: 87.4; MdA: 16.5; UCLA: 8.4; CAM deformity: 22%	5.5%	Hip subluxation: 5.5%; Implant failure: 5.5%; Heterotopic ossification: 16.7%; Implant removal: 22.2%	Good long-term results with low incidence of AVN and osteoarthritis but frequent revision surgery (FAI and implant removal). Procedure technically demanding.
Zuo et al., J Orthop Surg Res, 2020 [36](Retrospective)	21	13.2 y	20/1	1/20/0	0/0/21	2.6 y	Average HHS: 96.7; WOMAC: 95.4	0%	Implant failure: 4.8%;	Procedure technically demanding but safe and extremely valuable for restoring hip anatomy and preserving function in severe SCFE.
Ebert et al., J Orthop Surg Res, 2019 [35] (Retrospective)	15	12.9 y	15/0	0/8/7	0/0/15	3.8 y	Average HHS: 85.7; NHP: 0.91; VAS: 1.6; SDC: 27.8	26.7%	Hip subluxation: 13.3%; implant failure caused by AVN: 13.3%	Satisfactory results in most patients but, considering the risk of complications, the procedure is only indicated in severe chronic or acute on chronic SCFE.
Davis et al., JPO, 2019 [34] (Retrospective)	48	13.8 y (stable) 12.5 y (unstable)	17/31	31/17/0	N/A	2.9 y (stable), 2.3 y (unstable)	No data are reported regarding the final results except complications	29.4% (stable), 6.4% (unstable)	Hip subluxation: 17.6% (stable); Heterotopic ossification: 9.7% (unstable), 5.9% (stable); Hardware removal: 12.9% (unstable), 29.4% (stable)	Effective procedure in stable and unstable hips. Complication rate higher in stable hips. Caution in chronic stable SCFE
Sikora-Klak et al., JPO, 2019 [33] (Retrospective Comparative)	14	13.1 y	14/0	0/9/5	0/?/?	2.4 y	No data are reported regarding the final results except complications	28.6%	Significant limb length inequality: 7.1%	In consideration to the high incidence of AVN observed, the authors are against the procedure in stable, moderate or severe SCFE, preferring Imhauser osteotomy (AVN: 0%).
Lerch et al., Bone Joint J, 2019 [32] (Retrospective)	46	13 y	32/14	9/12/27	0/0/46	9 y	Average HHS: 94; HOOS: 91; MdA: 17; UCLA: 8; WOMAC: 4; CAM deformity: 7.5%	5%	Heterotopic ossification: 5%; Implant failure: 7.5%; Implant removal: 17.5%	High functional score at long-term follow-up with low rate of AVN observed only in unstable, acute on chronic slip. Secondary impingement deformities can develop and require further surgery.
Novais al, Int Orthop, 2019 [31] (Retrospective Comparative)	27	12.6 y	0/27	14/0/13	0/0/27	2.4 y	Heyman and Herdon outcomes: excellent or good: 67%	26%	Trochanteric screw breakage: 3.7%	The theoretical advantage of preserving blood supply reducing AVN rate was not observed. However, the authors observed better results in comparison to closed reduction and percutaneous pinning.
Masquijo et al., JPO, 2019 [30] (Retrospective Multicentric)	21	12 y	6/15	4/4/13	Mean preoperative value of slip angle: 59.1°	3.4 y	Average HHS: 76.3	28.6%	Superficial infection: 4.8%; Implant removal: 28.6%	Procedure technically demanding with a high rate of complications probably related to the learning curve. AVN more frequent in unstable hip
Persinger et al., JPO, 2019 [29](Retrospective)	31	12.4 y	0/31	31/0/0	N/A	2.4 y	Satisfactory results: 94%	6.4%	Mild heterotopic ossification: 6.7%; Implant failure: 3.2%; Implant removal: 6.4%	Procedure safe and effective for unstable SCFE. Low incidence of AVN and other complications. No cases of AVN in patients treated < 24 h.
Trisolino et al., JPO, 2018 [28] (Retrospective Comparative)	15	13.9 y	15/0	0/0/15	0/0/15	3.7 y	NAHS (total): 85.4	20%	Mild heterotopic ossification: 6.7%	Procedure restored the proximal femoral anatomy but there is a potential risk of AVN in comparison to SCFE treated by in situ fixation.
Ziebarth et al., CORR, 2017 [27] (Retrospective)	43	13 y	38/5	10/18/15	10/27/6	12 y	Average MdA: 17; Prevalence of limp: 0%; Positive Drehaman sign: 0%. Cumulative survivorship: 93%; Secondary impingement: 13%	0%	Refixation of the epiphysis: 9.3%; Reosteosyntesis of the greater trochanter: 2.3%; Implant removal: 20.9%	Procedure, when performed correctly, restored hip anatomy and hip function in stable, moderate or severe SCFE. No hips showed AVN or conversion to THA. Secondary impingement may persist in some hips that need further surgery.
Elmarghany et al., SICOT J, 2017 [26] (Prospective)	32	14 y	32/0	0/32/0	0/11/21	1.2 y	Average HHS: 96.2; MdA: 16.8; WOMAC: 3.3; Heyman and Herndon outcome: excellent or good 93.7%	9.3%	Postoperative deep infection: 3.1%; Revision for bad reduction: 3.1%	Procedure restored the normal proximal femoral anatomy, reducing the probability of secondary osteoarthritis and FAI.
Abdelazeem et al., Bone Joint J, 2016 [25] (Prospective)	32	14.3 y	32/0	0/32/0	0/10/22	2 y	Average HHS: 96.3; MdA: 16.8; WOMAC: 97	3.1%	Postoperative deep infection: 3.1%	Procedure safe and effective for stable SCCFE with high degree of slip.
Novais et al., CORR, 2015 [24] (Retrospective Comparative)	15	14 y	15/0	N/A	0/0/15	2.4 y	Heyman and Herdon outcomes: excellent/good: 60%	6.7%	Implant failure: 6.7%; Intraarticular pin penetration: 6.7%	Higher rate of excellent and results, with similar occurrence of complications when compared to SCFE treated by in situ pinning.
Upasani et al., JPO, 2014 [23] (Retrospective)	43	11.9 y	17/26	17/11/15	0/6/37	2.6 y	No data are reported regarding the final results but high complication rates are reported (> 40%)	23.2%	Femoral neck nonunion: 9.3%; Postoperative hip dislocation: 4.6%; Heterotopic ossification: 2.3%; Implant failure: 2.3%	Complication rate high. Presence of an expert surgeon during the procedure. AVN more frequent in unstable acute and acute on chronic SCFE (90%).
Souder et al., JPO, 2014 [22] (Retrospective Comparative)	17	12.2 y	10/7	N/A	N/A	1.3 y	AVN in 2/10 stable and in 2/7 unstable (Dunn); AVN in 0/64 stable and in 3/7 unstable (in situ pinning)	23.5%	Condrolysis: 5.9%; Implant failure: 5.9%	Attempts to anatomically reduce stable slips led to severe AVN in 20% of cases. Treatment of unstable slips remains problematic with high AVN rates whether treated by Dunn or in situ pinning.
Sankar et al., JBJS Am, 2013 [21] (Retrospective Multicentric)	27	12.6 y	0/27	27/0/0	0/6/37	1.8 y	Average HHS: 88 (no AVN), 60 (AVN); Satisfaction: 97.1% (no AVN), 65.8% (AVN); UCLA: 9.3 (no AVN), 5.9 (AVN)	25.9%	Implant failure: 14.8%	Procedure is able to restore anatomy and preserve hip function but AVN and implant complications may occur.
Madan et al., JBJS Br, 2013 [20] (Prospective)	28	12.9 y	11/17	9/11/8	0/0/28	3.2 y	Average HHS: 89.1; NAHS: 91.3	7.1%	Condrolysis: 3.6%	Procedure safe and reliable in patients with SCFE. ROM at final follow-up was nearly normal.
Massé et al., Hip Int, 2012 [19] (Retrospective)	20	14.3 y	18/2	N/A	8/4/8	2.0 y	Average HHS: 98.2; WOMAC: 0.6 (pain), 2.2 (function)	0%	Wire penetration in the hip joint: 5%; painful implant (removal): 5%;	The small number of technical complications appears favorable considering the surgical complexity of the procedure.
Huber et al., JBJS Br, 2011 [18] (Retrospective Multicentric)	30	12.2 y	27/3	3/?/?	3/17/10	3.8 y	Average HHS: 97.8; WOMAC: 5.9 (pain), 10.4 (stiffness), 5.7 function)	3.3%	Implant failure: 13.3%	Anatomical reduction can be achieved using this procedure with low risk of AVN. Implant failures may occur.
Slongo et al., JBJS Am, 2010 [17] (Retrospective)	23	11.9 y	20/3	0/9/14	N/A	2.4 y	Average HHS: 99; MdA: 17	8.7%	Wire penetration in the hip joint: 4.3%	Procedure minimizes secondary femoroacetabular Cam impingement and osteoarthritis. Complication rate low even in unstable SCFE.
Ziebarth et al., CORR, 2009 [16] (Retrospective Multicentric)	40	11.9 y	27/13	11/29/?	0/16/19 (no information on 5 hips)	2.6 y	Average HHS: 99.6; MdA: 17.8; WOMAC: 1.2 (pain), 3 (function)	0%	Heterotopic ossification: 2.5%; Residual impingement: 2.5%; Delayed union: 7.5%; Implant failure: 7.5%;	Acceptable complication rate. Procedure reproducible for full correction of moderate to severe SCFE with open physis.

From the analysis of our selected literature, Dunn osteotomy modified by Ganz, performed by an experienced surgeon, allows for anatomical reduction of moderate or severe SCFE with a low incidence of AVN. In mild SCFE, there is no consensus on the use of this technique, since several authors prefer to perform an in situ fixation and treat possible FAI later on by hip arthroscopy.

The majority of the reported studies have a short-term follow-up, which ranges from 1.2 to 3.8 years. Only three studies reported long term follow-up of 12, 9 and 9.4 years respectively. These studies seem to show the absence of degenerative hip osteoarthritis at follow-up in patients treated by this technique.

Treatment of SCFE is still controversial, especially in moderate and severe forms [39]. It is also related to the mode of onset of the disease, if acute or chronic or acute on chronic, and to the stability of the capital epiphysis on the femoral neck. According to our systematic review we believe that Dunn osteotomy modified by Ganz should be strongly considered in moderate and severe forms of SCFE, because it allows an anatomical reduction of the femoral head sparing vascular supply in the majority of the cases. There is no general consensus on using this technique in mild SCFE. The learning curve may be long and should be taken into consideration.

Generally, pinning in situ represents the treatment of choice for mild SCFE with the goal to prevent further slip progression [40], although some authors have reported mild forms treated by Dunn osteotomy modified by Ganz. In our series of selected papers, four studies reported an overall number of 29 patients affected by mild SCFE treated by osteotomy according to the Ganz surgical approach [18, 19, 27, 37]. Two studies, justify this option of treatment arguing that even mild forms of SCFE may cause secondary FAI which leads to late hip osteoarthritis [19, 27]; both reported an overall number of 63 patients surgically treated with no cases of AVN. Huber et al. [18] suggested an intraoperative inspection of the slipping after the hip dislocation, before making the final decision whether to perform only in situ fixation or osteotomy. They reported three cases classified as mild SCFE in which the displacement was more evident than expected and needed a repositioning of the capital epiphysis. The last study that reported eight hips with a mild slip treated by osteotomy, reported in their limitations that all patients were surgically treated regardless of the severity, stability and chronicity of the slip [37]. We believe, in agreement with the majority of the authors, that, considering the risk of AVN that may still occur after surgery even in patients treated by expert hip surgeons, the preferred option of treatment of mild SCFE might be in situ fixation, treating in a second time possible FAI by arthroscopic trimming of the metaphysis [41].

On the contrary, in moderate and severe forms of SCFE, there is a high risk of degenerative joint disease caused by the consequent deformity of the femoral head. Therefore, a surgical correction by anatomical repositioning of the capital epiphysis on the femoral neck, should be the treatment of choice, even with an incidence of AVN > 20% [21–23, 28, 30, 31, 33–35]. Dunn osteotomy performed with Ganz approach represents the only surgical procedure for restoring the correct anatomy of the proximal part of the femur without stretching the retinaculum vessels, however the operation is still technically demanding. The majority of the studies in spite of a high rate of AVN, are in favor of this procedure, although there is disagreement regarding the indications in relation to the type of SCFE [21–23, 30, 31, 34, 35]. Davis et al. [34], reported better results in unstable hips in which the AVN rate was 6.4% in comparison to 29.4% observed in stable hips. On the contrary, other authors [22, 23] reported better results in stable SCFE with AVN more frequently observed in unstable acute and acute on chronic slips. Novais et al. [24], in a series of severe unstable hips, in spite of an incidence of 26% of AVN observed after Dunn procedure, reported better results in comparison to closed reduction and percutaneous pinning. Finally, some authors [23, 30] related their high incidence of AVN to the surgeon, stressing the importance of the learning curve and suggest the presence of an expert orthopaedist, reporting a statistically significant association between surgeon and incidence of complications [23].

The remaining 14 papers reported a low incidence of AVN, from 0 to 9.3%. The total number of operated hips were 409, the Harris Hip Score ranged from 81.8 to 99.6 points, while the Merle d’Aubigné score ranged from 16.5 to 17.8 points. All these authors recommend Dunn procedure modified by Ganz in moderate and severe SCFE, because when it is performed correctly, it restored hip anatomy and function. They considered this procedure safe, reliable and reproducible, and recommend it as the first choice of treatment, although it is technically demanding and requires an expert surgeon. The higher incidence of AVN was observed in unstable acute on chronic SCFE [32].

Regarding the other complications, in the majority of the selected studies (17 papers) [16–19, 21–24, 27, 29–32, 34–37] the authors performed implant removal for implant failure, related to AVN or different reasons. The incidence of implant removal ranged from 2.3 to 29.48%. In five papers [23, 34, 35, 37, 38] the authors observed hip instability with an incidence from 3.3 to 17.6% that required a second operation. This complication, as reported by some authors [42] may be directly related to SCFE such as damages of the acetabular labrum or the acetabular cartilage, causes not related to SCFE (acetabular orientation or poor quality of the soft tissues) and causes related to the surgical operation. They suggest to test “the congruity and stability of the hip during the surgical procedure and preferably treat this complication during the same period of anesthesia”.

Heterotopic ossifications are also described in seven papers [16, 23, 28, 29, 32, 34, 37] with an incidence from 2.3 to 16.7%, but in the majority of the cases they were completely asymptomatic. Other complications such as, deep infections [25, 26], femoral neck nonunion [23], condrolysis [20, 22] or significant limb length inequality [33] are extremely rare and reported by one or two papers with a low incidence.

Regarding the stability of the SCFE, many papers report their results of stable and unstable hips together, without differentiating them in terms of clinical results and complications, although the majority of the hips in these studies were stable (259 stable hips vs 121 unstable hips reported in 13 papers) [16–20, 22, 23, 27, 30, 32, 36–38]. Davies et al. [34], reported a cohort of 48 SCFE (17 stable and 31 unstable), differentiating their results according to the hip stability. They observed most frequent complications in stable hips (AVN: 29.4% vs 6.4%, hip instability: 13.3% vs 0%), but they concluded that, although the complications are higher in stable hips, the surgical procedure is effective in both groups. Six papers [24–26, 28, 33, 35] reported only patients with stable hips (123 hips), three of them had an incidence of AVN > 20%, while the other three, a low incidence. The remaining three papers [21, 29, 31] reported only patients with unstable hips (85 hips), with a high incidence of AVN in two studies (about 26%). These data confirm that Dunn osteotomy modified by Ganz is more frequently used in stable SCFE and the higher risk of AVN seems to be in unstable hips.

The majority of the reported studies have a short-term follow-up, which ranges from 1.2 to 3.8 years. Only three studies reported long term follow-up of 12, 9 and 9.4 years respectively [27, 32, 37]. Ziebarth et al. [27] reported a 12 year long term follow-up study, analyzing 43 hips affected by SCFE operated according to modified Dunn procedure. The authors observed in > 90% of cases an excellent or good result with no progression of osteoarthritis. No hips showed signs of AVN at the latest follow-up. Only four hips showed progression of osteoarthritis between 10 and 17 years follow-up but no patient needed a total hip arthroplasty. No difference was found in survivorship between mild, moderate and severe slips. Lerch et al. [32] reported a 9 years follow-up retrospective study in 46 hips with severe SCFE treated by modified Dunn procedure. They reported a low risk of progression of hip osteoarthritis and an incidence of 5% of AVN observed in two patients with an unstable acute on chronic slip that needed further surgery. FAI was observed in three patients, but only in one case the reduction was incomplete. The other two developed a later deformity related to the remodeling of the capital epiphysis. Passaplan et al. [37] reported a 9.4 year follow-up in which they analyzed 18 SCFE surgically treated by modified Dunn procedure. They reported a low rate of osteoarthritis and observed AVN only in two cases that were slightly symptomatic, with a good long-term clinical outcome. An asymptomatic osteoarthritis grade I was seen in both hips. Moreover, they observed five hips with an anterior impingement but only two of them had a pathological alfa angle on the axial view.

The majority of the papers are retrospective studies without a control group. Only five studies reported comparative results of Dunn osteotomy versus in situ pinning [22, 24, 28, 31] or Imhauser osteotomy [33]. There is general agreement that in severe stable hips, AVN is more frequent with modified Dunn osteotomy in comparison to pinning in situ (20% vs 0%) [22, 24, 28]. On the contrary, in unstable hips, similar or better results using modified Dunn procedure are reported [22, 31]. Only one paper compared Dunn procedure with Imhauser osteotomy; Sikora-Klak et al. [33] reported better results with Imhauser osteotomy in comparison to modified Dunn procedure. Some recent papers suggested to improve Imhauser osteotomy by performing a neck osteochondroplasty through a surgical dislocation approach. They considered this procedure safe and effective in 62 hips with severe stable SCFE after a mean follow-up of 4.1 years [43–45]. However, in a previous 24 years long term follow-up study in which SCFE were treated by Imhauser osteotomy, the incidence of osteoarthritis was high (45%) [46].

In the few prospective studies [20, 25, 26] the authors reported that Dunn osteotomy represents a safe and effective procedure that restored a normal proximal femoral anatomy with a nearly normal range of motion of the operated hip, reducing the probability of secondary osteoarthritis and FAI.

**Fig. 2:**
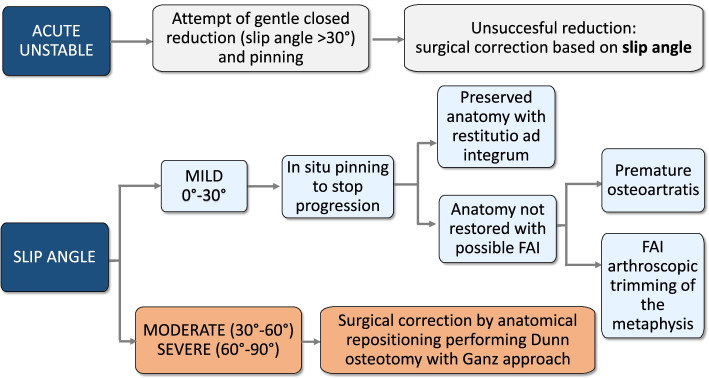
Flow chart of surgical treatment according to clinical and radiographic classifications

Based on our review, we propose a flow chart of surgical treatment according to clinical and radiographic classifications (Fig. 2).

There are some limitations with this systematic review. Most of the studies included on the Dunn osteotomy modified by Ganz are retrospective, and as such, the risk of selection and information bias is high. Long term multicentric RCT are needed to better evaluate the efficacy of this treatment.

## Conclusions

In conclusion, we believe that Dunn osteotomy modified by Ganz should be the preferred method to treat moderate or severe SCFE. The few papers with long term follow-up, reported no progression of hip osteoarthritis, however, since the patients are adolescents at surgery, more time is needed to evaluate the effectiveness of this treatment. It is still debated if better results are obtained in stable or unstable SCFE and further studies should focus on determining the importance of stability in the prognosis of this procedure. In mild SCFE, we believe that the possible risk of AVN of this procedure does not justify its use, since resulting FAI can be treated further down the line through hip arthroscopy.

## Data Availability

The datasets used and/or analyzed in the current study are available from the corresponding author on reasonable request.

## Abbreviations

AVN: Avascular Necrosis
FAI: Femoro-Acetabular Impingement
PICO: Population Intervention Comparison Outcome
PRISMA: Preferred Reporting Items for Systematic Reviews and Meta-Analyses
RCT: Randomized Control Trial
SCFE: Slipped Capital Femoral Epiphysis
